# Loss of G protein pathway suppressor 2 in human adipocytes triggers lipid remodeling by upregulating ATP binding cassette subfamily G member 1

**DOI:** 10.1016/j.molmet.2020.101066

**Published:** 2020-08-13

**Authors:** Serena Barilla, Ning Liang, Enrichetta Mileti, Raphaëlle Ballaire, Marie Lhomme, Maharajah Ponnaiah, Sophie Lemoine, Antoine Soprani, Jean-Francois Gautier, Ez-Zoubir Amri, Wilfried Le Goff, Nicolas Venteclef, Eckardt Treuter

**Affiliations:** 1 Department of Biosciences and Nutrition, Karolinska Institute, 14183 Huddinge, Sweden; 2 Centre de Recherche des Cordeliers, Inserm, University of Paris, IMMEDIAB Laboratory, F-75006, Paris, France; 3 Inovarion, Paris, France; 4 ICANalytics Lipidomic, Institute of Cardiometabolism and Nutrition (ICAN), Paris, France; 5 École Normale Supérieure, PSL Research University, Centre National de la Recherche Scientifique (CNRS), Inserm, Institut de Biologie de l'École Normale Supérieure (IBENS), Plateforme Génomique, Paris, France; 6 Department of Digestive Surgery, Générale de Santé (GDS), Geoffroy Saint Hilaire Clinic, 75005, Paris, France; 7 Lariboisière Hospital, AP-HP, Diabetology Department, University of Paris, Paris, France; 8 University of Côte d’Azur, CNRS, Inserm, iBV, Nice, France; 9 Sorbonne University, Inserm, Institute of Cardiometabolism and Nutrition (ICAN), UMR_S1166, Hôpital de la Pitié, Paris, F-75013, France

**Keywords:** Adipocytes, Adipogenesis, Hypertrophy, Obesity, Type 2 diabetes, GPS2, ABCG1

## Abstract

**Objective:**

Adipogenesis is critical for adipose tissue remodeling during the development of obesity. While the role of transcription factors in the orchestration of adipogenic pathways is already established, the involvement of coregulators that transduce regulatory signals into epigenome alterations and transcriptional responses remains poorly understood. The aim of our study was to investigate which pathways are controlled by G protein pathway suppressor 2 (GPS2) during the differentiation of human adipocytes.

**Methods:**

We generated a unique loss-of-function model by RNAi depletion of GPS2 in human multipotent adipose-derived stem (hMADS) cells. We thoroughly characterized the coregulator depletion-dependent pathway alterations during adipocyte differentiation at the level of transcriptome (RNA-seq), epigenome (ChIP-seq H3K27ac), cistrome (ChIP-seq GPS2), and lipidome. We validated the *in vivo* relevance of the identified pathways in non-diabetic and diabetic obese patients.

**Results:**

The loss of GPS2 triggers the reprogramming of cellular processes related to adipocyte differentiation by increasing the responses to the adipogenic cocktail. In particular, GPS2 depletion increases the expression of *BMP4*, an important trigger for the commitment of fibroblast-like progenitors toward the adipogenic lineage and increases the expression of inflammatory and metabolic genes. GPS2-depleted human adipocytes are characterized by hypertrophy, triglyceride and phospholipid accumulation, and sphingomyelin depletion. These changes are likely a consequence of the increased expression of ATP-binding cassette subfamily G member 1 (*ABCG1*) that mediates sphingomyelin efflux from adipocytes and modulates lipoprotein lipase (LPL) activity. We identify *ABCG1* as a direct transcriptional target, as GPS2 depletion leads to coordinated changes of transcription and H3K27 acetylation at promoters and enhancers that are occupied by GPS2 in wild-type adipocytes. We find that in omental adipose tissue of obese humans, *GPS2* levels correlate with *ABCG1* levels, type 2 diabetic status, and lipid metabolic status, supporting the *in vivo* relevance of the hMADS cell-derived *in vitro* data.

**Conclusion:**

Our study reveals a dual regulatory role of GPS2 in epigenetically modulating the chromatin landscape and gene expression during human adipocyte differentiation and identifies a hitherto unknown GPS2-ABCG1 pathway potentially linked to adipocyte hypertrophy in humans.

## Highlights

• GPS2 depletion in human adipose-derived mesenchymal stem cells increases expression of adipogenic genes, including BMP4.
• Loss of GPS2 leads to coordinated changes of epigenome and transcriptome during human adipocyte differentiation.
• Loss of GPS2 upregulates ABCG1 and LPL and induces lipidome remodeling including sphingomyelin depletion.
• The GPS2-ABCG1 pathway contributes to adipocyte hypertrophy.
• GPS2 and ABCG1 levels in omental adipose tissue inversely correlate with type 2 diabetes in obese humans.

## 1 Introduction

The rising prevalence of obesity and its strong association with comorbidities such as insulin resistance and type 2 diabetes has increased interest in adipose tissue biology and its therapeutic potential. Obesity is recognized as a pathological condition characterized by body fat accumulation with expansion of adipose depots in response to high caloric intake. Adipose tissue plasticity plays a critical role in systemic metabolic homeostasis and the ability of adipocytes to effectively store lipids, thereby protecting other tissues such as muscle and liver from lipotoxicity [1]. Adipose tissue enlargement is achieved either via hyperplasia, that is, the increased number of adipocytes through differentiation of resident progenitors, or via hypertrophy, that is, the increased size of adipocytes due to lipid accumulation [2]. The balance between these two processes has a significant impact on metabolic health. Hypertrophy has been shown to be a relevant marker for the development of type 2 diabetes in the context of obesity [3]. Enlarged adipocytes seem to correlate with adipose tissue dysfunction and increased systemic insulin resistance, while smaller adipocytes seem to counteract obesity-associated diabetes [4,5].

Adipogenesis is a process in which fibroblast-like progenitors become committed to the adipogenic lineage, accumulate triglycerides, and differentiate into mature lipid-filled adipocytes. The ability to recruit new adipocytes through adipogenesis in the setting of nutrient excess is a critical factor for metabolically healthy adipose tissue remodeling. Deregulation of adipogenesis in response to overnutrition has consequences on adipose tissue expandability and leads to pathologic adipose tissue remodeling coupled with insulin resistance [6]. Multiple studies, in particular using the mouse 3T3-L1 preadipocyte cell line as a standard model, have elucidated mechanisms underlying adipogenesis and uncovered complex networks of transcription factors that orchestrate this process, including the master regulators peroxisome proliferator-activator receptor (PPAR) γ and members of the CCAAT-enhancer-binding protein (C/EBP) family including C/EBPα [[7], [8], [9], [10], [11]]. Considering that there could be species differences regarding the key components and mechanisms underlying adipogenesis and adipocyte biology, there is a recognized need for investigations using human adipocytes. Among the most relevant models are human primary adipocyte cultures from different origins [[12], [13], [14], [15]] and immortalized cell models derived from human white adipose tissue. The latter include SGBS cells derived from stromal cells of an infant with Simpson-Golabi-Behmel (SGBS) syndrome [16] and human multipotent adipose-derived stem (hMADS) cells derived from adipose tissue of healthy male young donors [17]. hMADS cells represent a particularly relevant human cell model as they retain many molecular and functional properties of human white adipocytes, including browning, with a high self-renewal capacity and can differentiate into mature adipocytes and osteocytes [18,19]. Despite these advances, the role of transcription factor-associated coregulators in human adipogenesis is currently not well understood. Additionally, it is unknown which coregulators are potentially involved in transducing regulatory signals into epigenome alterations and transcriptional responses linked to adipose tissue hypertrophy in humans.

Evidence is emerging that subunits of a fundamental corepressor complex may be involved in the aforementioned processes. This complex, commonly known as the HDAC3 corepressor complex, contains the core subunits histone deacetylase 3 (HDAC3), nuclear receptor corepressor (NCoR, alias NCOR1), silencing mediator of retinoid and thyroid hormone receptors (SMRT, alias NCOR2), transducin β-like proteins TBL1 and TBLR1, and G protein pathway suppressor 2 (GPS2) [[20], [21], [22]]. Several mouse models have revealed first insights into the role of these core subunits in adipose tissue but also demonstrated selective phenotypes, in part opposing each other, that remain difficult to interpret. For example, adipocyte-specific NCoR knockout mice display increased adiposity and insulin sensitivity [23], while mutant SMRT mice show hypertrophic adipose tissue and insulin resistance [24,25]. Notably, a genome-wide investigation using mouse 3T3-L1 cells supports a role of SMRT as a gatekeeper of adipogenesis in mice [26], consistent with earlier data showing that NCoR and SMRT act as corepressors of PPARγ to inhibit adipogenesis in the 3T3-L1 cell model [27]. Moreover, two independent studies generated adipocyte-specific HDAC3 knockout mice but reported opposite effects on the browning of white adipose tissue [28,29]. Also, loss of TBLR1 in adipocytes increases adiposity and induces insulin resistance in mice, likely acting on lipolysis [30].

Multiple studies have particularly pinpointed the involvement of the subunit GPS2 in adipocyte biology. Obese and diabetic mouse models overexpressing or lacking GPS2 specifically in adipocytes identified a crucial role of GPS2 in controlling inflammatory responses [31,32]. Our work revealed that the anti-inflammatory role of GPS2 is conserved in human adipocytes [12]. In this study, we also demonstrated that the expression of GPS2 in white adipocytes of obese subjects is reduced, which involves transcription factor TWIST1, and negatively correlated to the expression of proinflammatory genes [12]. Investigations in mouse 3T3-L1 cells have suggested that GPS2 regulates lipid metabolism via modulating PPARγ at the promoters of lipolytic enzymes [33], which involves KDM4 histone demethylase and is consistent with our earlier findings [34]. GPS2 may also play a cytoplasmic role by regulating insulin signaling via AKT ubiquitination [35]. Our recent work discovered that GPS2 controls adipocyte hypertrophy in mice by repressing transcription factor HIF1α [36]. We found that dysregulation of the GPS2-HIF1α axis disrupts mitochondrial activity and predisposes to adipose tissue expansion and a pro-diabetic status. In summary, while these studies support that GPS2 controls a number of metabolic and inflammatory pathways in mature adipocytes in mice, its functions in human adipocytes and adipogenesis have not yet been addressed.

In this study, we investigated the role of GPS2 in human adipose progenitor cells and the consequences of GPS2 depletion on adipocyte differentiation and metabolism using the hMADS cell model. We characterized the loss of GPS2-triggered alterations by comparing transcriptome (RNA-seq), epigenome (ChIP-seq H3K27ac), cistrome (ChIP-seq GPS2), and lipidome, along with determining phenotypical alterations. This enabled us to identify a hitherto unrecognized dual role of GPS2 during adipogenesis. In the early stage, GPS2 inhibits the commitment of precursor cells toward the adipocyte lineage. At the late stage, GPS2 inhibits sphingolipid efflux via repressing *ABCG1* expression and thereby plays a pivotal role in controlling the hypertrophic phenotype in human adipocytes.

## 2 Materials and methods

### 2.1 Human tissue samples

Visceral adipose tissue (VAT) biopsies were obtained from different populations admitted to the Geoffroy Saint-Hilaire Clinic (Paris, France). Clinical and anthropometric data are detailed in Supplementary Table 1. This study was conducted in accordance with the Helsinki Declaration as previously described [32,36]. The Ethics Committee of CPP Ile-de-France approved the clinical investigations, and written informed consent was obtained from all of the individuals. VAT biopsies were obtained from obese subjects during bariatric surgery.

### 2.2 hMADS cell culture and differentiation

The hMADS cells were multipotent stem cells isolated from prepubic fat pads of a 4-month-old healthy male [17,18]. The hMADS cells had a normal karyotype and were used between passages 12 and 24. The hMADS cells were maintained in DMEM (Lonza, low glucose) supplemented with 10% fetal calf serum (Eurobio), 15 mM of Hepes (Lonza), 2 mM of l-glutamine (Lonza), 1% penicillin/streptomycin, and 2.5 ng/ml of hFGF2 (Peprotech). Two days post–confluence (day 0), the cells were induced to differentiate in DMEM/Ham's F12 (Lonza) media supplemented with 1 μM of dexamethasone, 500 μM of 3-isobutyl-1-methylxanthine (IBMX), 5 μg/ml of insulin, 10 μg/ml of transferrin, and 0.2 nM of triiodothyronine (T3). After two days of induction, the dexamethasone and IBMX were omitted from the media, and 100 nM of rosiglitazone was added until day 9. The differentiation was continued until day 14, when full white adipocytes were developed.

### 2.3 Lentiviral short hairpin RNA (shRNA) production and transfection of hMADS cells

For GPS2 depletion via RNA interference, shRNA oligos (pre-designed by Dharmacon) targeting mRNAs encoding GFP (targeting sequence GCAAGCTGACCCTGAAGTTCA) or GPS2 (targeting sequence CCAGAGCATGGACAATTCCAA) were cloned into pLKO.1 TCR vector (Addgene). *In silico* blasting was conducted using the Basic Local Alignment Search Tool (BLAST). The target sequence for shGPS2 was located in exon 7. Lentiviral particles were produced in human embryonic kidney (HEK) 293FT cells using psPAX2 (Addgene) packaging plasmid and pMD2.G (Addgene) envelope plasmid. Then 70% confluent hMADS preadipocytes were transfected with lentivirus shRNA particles in fresh media containing 8 μg/ml of polybrene (Sigma–Aldrich). 24 h after transfection, cells were selected using puromycin for 4 days. Mature hMADS adipocytes (day 9 after induction of differentiation) were incubated for 24 h with lentiviral media containing 8 μg/ml polybrene (Sigma–Aldrich) before the medium was changed to normal differentiation media.

### 2.4 Quantitative reverse transcription PCR (RT-qPCR)

Total RNA was extracted from hMADS cells using TRIzol reagent (Thermo Fisher Scientific) and an RNeasy RNA Mini kit (Qiagen). Complementary DNAs were synthesized using M-MLV Reverse Transcriptase (Life Technologies). RT-qPCR was conducted using an ABI Prism 7500 PCR system (Applied Biosystems). Ribosomal protein lateral stalk subunit P0 (RPLP0) was used for normalization to quantify the relative mRNA expression levels. Relative changes in mRNA expression were calculated using the comparative cycle method (2^−ΔΔCt^). The primers are listed in Supplementary Table 2.

### 2.5 RNA-seq

RNA was extracted as previously described. The RNA quality was assessed using a 2200 TapeStation Instrument (Agilent). For library preparation and sequencing, 8 ng of RNA was processed at the EMBL Genomic Core Facility (Heidelberg, Germany) using standard protocols and sequenced in an Illumina HiSeq 2000. For human tissue samples, library preparation and Illumina sequencing were conducted at the Ecole Normale Superieure Genomic Core Facility (Paris, France). Messenger (polyA+) RNAs were purified from 400 μg of total RNA using oligo (dT). Libraries were prepared using a strand-specific RNA-Seq library preparation TruSeq Stranded mRNA kit (Illumina). Then 75 bp single read sequencing was conducted on a NextSeq 500 (Illumina). Preprocessed reads were aligned to the hg38 transcriptome HISAT2 program, and Hypergeometric Optimization of Motif EnRichment (HOMER, http://homer.salk.edu/) was used to create the tag directory and count the tags in all of the exons. Raw tag counts were imported into R and Bioconductor, edgeR package [37] was used to determine differential gene expression, and the clusterProfiler package [38] or self-organizing map [39] was used for the clustering.

### 2.6 Chromatin immunoprecipitation (ChIP)

ChIP experiments were conducted as previously described [9] with some minor modifications. Briefly, hMADS cells were double crosslinked with 2 mM of disuccinimidyl glutarate (DSG) for 30 min, followed by 1% formaldehyde for 10 min. The reaction was stopped with glycine at a final concentration of 0.125 M for 10 min, followed by the addition of ChIP nuclear isolation buffer (1% SDS, 50 mM of Tris–HCl, and 20 mM of EDTA). Isolated nuclei were resuspended in ChIP buffer (0.1% SDS, 1% Triton X-100, 0.15 M of NaCl, 1 mM of EDTA, and 20 mM of Tris–HCl) and subsequently sonicated for 3 × 10 cycles (30 s on/off) in a Pico Bioruptor (Diagenode) to generate 0.2–0.5 kb DNA fragment sizes. Chromatin from 10–20 × 10^6^ cells was used for histone marks and 20–30 × 10^6^ cells for GPS2 ChIP. Each lysate was immunoprecipitated with the following antibodies: anti-H3K27ac (Abcam, ab4729, 1 μg) and anti-GPS2 (custom-made [34], 4 μg). Following 3 h of rotation at 4 °C, 25 μl of protein A Dynabeads (Invitrogen) were added and the samples were incubated overnight at 4 °C with rotation. The next day, the samples were washed twice with IP wash buffer 1 (1% Triton, 0.1% SDS, 150 mM of NaCl, 1 mM of EDTA, 20 mM of Tris, pH 8.0, and 0.1% sodium deoxycholate [NaDOC]), twice with IP wash buffer 2 (1% Triton, 0.1% SDS, 500 mM of NaCl, 1 mM of EDTA, 20 mM of Tris, pH 8.0, and 0.1% NaDOC), twice with IP wash buffer 3 (0.25 M of LiCl, 0.5% NP-40, 1 mM of EDTA, 20 mM of Tris, pH 8.0, and 0.5% NaDOC), and once with IP wash buffer 4 (10 mM of EDTA and 200 mM of Tris, pH 8), all at 4 °C. Immune-bound chromatin was eluted in elution buffer (1% SDS and 0.1 M of NaHCO_3_) and de-crosslinked by adding NaCl to a final concentration of 0.2 M at 65 °C O/N. After RNAse A/T (Fermentas) and proteinase K (Fermentas) treatment, the immunoprecipitated DNA was purified using a ChIP DNA Clean and Concentrator Capped Zymo-Spin I (Zymo Research) purification kit.

### 2.7 ChIP-seq

For ChIP-seq library preparation and sequencing, 2–10 ng of ChIPed DNA was processed using a Rubicon ThruPLEX DNA-seq kit (Takara) using standard protocols and sequenced in a NextSeq 550 (75SE reads, BEA Core Facility, Karolinska Institute, Sweden). The ChIP-seq data were computationally analyzed using resources provided by SNIC through Uppsala Multidisciplinary Center for Advanced Computational Science (UPPMAX) under Project SNIC 2018/8–122. The analysis was conducted as previously described [32]. Sequencing files (FASTQ files) provided by the BEA Core Facility (Karolinska Institute, Sweden) and raw data from the published ChIP-seq data (PPARγ: GSE115421; C/EBPα: GSE24326) were aligned to the GRCh38 version of the human reference genome using Bowtie 2 [40]. Peaks were identified using the HOMER package [41]. Peak heights were normalized to the total number of uniquely mapped reads and displayed in the Integrative Genomics Viewer (IGV) [42] as the number of tags per 10 million tags. The sequences found in GPS2 peaks were subjected to motif analysis to identify potential transcription factor-binding sites using HOMER with the IMAGE motif database [43]. To statistically analyze the peaks, the raw tag counts were imported into R and Bioconductor, and the edgeR package was used to identify potential differential binding sites [37].

### 2.8 Western blotting

Samples were lysed in RIPA buffer supplemented with protease and phosphatase inhibitors, diluted to a concentration of 20 μg of protein, and heated at 98 °C for 10 min. The proteins were separated by SDS-PAGE electrophoresis and transferred to PVDF or nitrocellulose membranes (Amersham International). The membranes were incubated for 1 h in blocking reagent (SuperBlock T20 (PBS) blocking buffer, Thermo Fisher Scientific), and primary antibody was incubated overnight at 4 °C in the blocking solution. The antibodies and their concentrations were as follows: anti-GPS2 (custom-made against N-terminus or C-terminus, respectively, of human GPS2 [34], 1:3000), anti-HSP90 (Santa Cruz, sc-7947; 1:2000), anti-ABCG1 (Novus, NB400-132; 1:500), and anti-β-ACTIN (Abcam, ab8226; 1:30,000). After several washes in PBST (PBS with 0.05% Tween 20), horseradish peroxidase (HRP)-labeled secondary antibodies (1:5000) were incubated for 1 h at room temperature in PBST. The membranes were developed with ECL Western blotting substrate (BioRad, 1705061). For the immunoblotting, IRDye 800CW-conjugated donkey anti-rabbit and IRDye 680RD-conjugated donkey anti-mouse (Li-Cor) secondary antibodies were incubated for 1 h at room temperature in blocking reagent. Detection was conducted using an Odyssey infrared imaging system (Li-Cor).

### 2.9 Co-immunoprecipitation

HEK293 (ATCC, CRL-1573) cells were co-transfected with pcDNA3-HA-GPS2 (encoding human GPS2 WT amino acid 1–327) and expression plasmids for the indicated Flag-tagged transcription factors. The cells were lysed 48 h after transfection, and the lysate was incubated with anti-Flag (Sigma–Aldrich, F7425) coupled with protein A magnetic beads for 3 h at 4 °C (previously 15 μl beads were incubated with 2 μg of antibody for 2 h at 4 °C). The beads were washed with lysis buffer five times and eluted at 98 °C for 10 min. The eluted sample was loaded in an acrylamide gel by following the Western blotting protocol and blotted with anti-Flag or anti-HA. Whole-cell lysis was used as the input. C/EBP plasmids were kindly provided by Susanne Mandrup (University of Southern Denmark, Denmark), and PPAR plasmids were gifts from Karolien De Bosscher (University of Gent, Belgium). The c-JUN and c-FOS cDNAs were obtained from the Karolinska Institute's High Throughput Center (KHTC) and cloned into pcDNA3.1-3XFlag-V5 expression vector which were kindly provided by Jussi Taipale (Karolinska Institutet, Sweden).

### 2.10 Quantification of lipid content

Quantification of lipids was conducted with Oil Red O staining. Differentiated cells were fixed with 10% formalin for 5 min and stained with Oil Red O (0.5% in isopropanol diluted 6:4 in water) for 15 min. Microscopy images were obtained using a 10× phase-contrast object on a Zeiss Axioskop 40 microscope. Oil Red O was quantified by eluting the color in isopropanol and measuring the absorbance at 500 nm. Accumulation of intracellular lipids was analyzed with BODIPY staining. Adipocytes were fixed with 4% paraformaldehyde for 10 min and stained with BODIPY (1 mg/ml diluted 1:5000) and DAPI (1:5000). The lipids and number of cells were quantified with CellInsight CX5 High-Content Screening Platform (Thermo Fisher Scientific). The lipid droplet amount, size, and cell number were analyzed using the Spot detector algorithm in HCS Studio software (Thermo Fisher Scientific). Lipid per cell was calculated by the ratio between the number of lipid droplets detected by BODIPY staining and the number of cells detected with the DAPI staining. The lipid droplet dimensions were calculated by the ratio between the total area of the lipid droplets detected by the BODYPI staining and the number of lipid droplets counted. Lipid-loaded cells were calculated by the percentage of BODIPY positive and the total number of cells. Immunofluorescence imaging was conducted using Nikon A1R and A1+ imaging systems (Nikon Corporation, Japan). The images were analyzed using NIS elements (Nikon Corporation, Japan).

### 2.11 Lipidomics

Cell pellets were extracted according to a modified Bligh and Dyer method [44]. The pellets were supplemented with internal standard and lipids extracted with 1.2 ml methanol/chloroform (2:1 v/v) in the presence of antioxidant BHT and 310 μl of HCl 0.01 N. Phase separation was triggered by the addition of 400 μl of chloroform and 400 μl of water. Extracted lipids were dried and resuspended in 40 μl of LC/MS solvent. The lipids were quantified by LC-ESI/MS/MS using a Prominence UFLC and QTrap 4000 mass spectrometer. Phospholipids and sphingolipids were quantified in the positive-ion mode. Samples were injected into a Kinetex HILIC 2.6 μm 2.1 × 150 mm column. Mobile phases consisted of water and acetonitrile containing ammonium acetate and acetic acid. Lipid species were detected using scheduled multiple reaction monitoring (sMRM). A total of 251 lipid species from 12 lipid subclasses (LPC, LPE, PC, PE, PEp, PI, PG, PS, PA, DHC, ceramides, and SM) were quantified. Isotopic contribution to MRM signals was corrected based on the approach of Ejsing et al. [45] using the open access R script (enviPat, GPLv2, Martin Loos and Christian Gerber). Batch effect and signal drift were corrected using the LOESS approach on the Workflow4Metabolomics open source tool. Data were expressed as mol% of total analyzed lipids.

### 2.12 Cholesterol efflux assay

Cholesterol efflux was determined using a cholesterol efflux fluorometric assay kit (BioVision) according to the manufacturer's protocol. Mature adipocytes were incubated overnight with labeling reagent. Then the labeling reagent was removed and the cellular cholesterol efflux to 20 μg/ml of free ApoA-1 (Sigma) or 50 μg/ml of HDL phospholipid (Sigma) was assayed in SILAC advanced medium (Gibco) for 5 h. Cellular and medium fluorescence were measured at Ex/Em = 485/523 nm.

### 2.13 LPL activity measurement

LPL activity was determined with a 50 μl aliquot of culture medium after 30 min of treatment with 50 units of heparin using an LPL activity assay kit (Sigma) according to the manufacturer's instructions. Intracellular total lipase activity was measured using a lipase activity assay kit III (Sigma).

### 2.14 Statistical analysis

All of the experiments were biological replicates that were repeated at least two times in the lab. All of the statistical tests were conducted using GraphPad Prism 6.0b (GraphPad Software, Inc., La Jolla, CA, USA), and all of the data are represented as mean ± SD. Statistical analysis was conducted using Student's unpaired t-test. All of the statistical tests were two-tailed, and *p* < 0.05 was considered statistically significant.

### 2.15 Sequencing data availability

The RNA-seq and ChIP-seq data were deposited at NCBI Gene Expression Omnibus (GEO) under accession number GSE152517.

## 3 Results

### 3.1 GPS2 depletion in preadipocytes increases transcription of genes involved in adipocyte commitment

**Figure 1:**
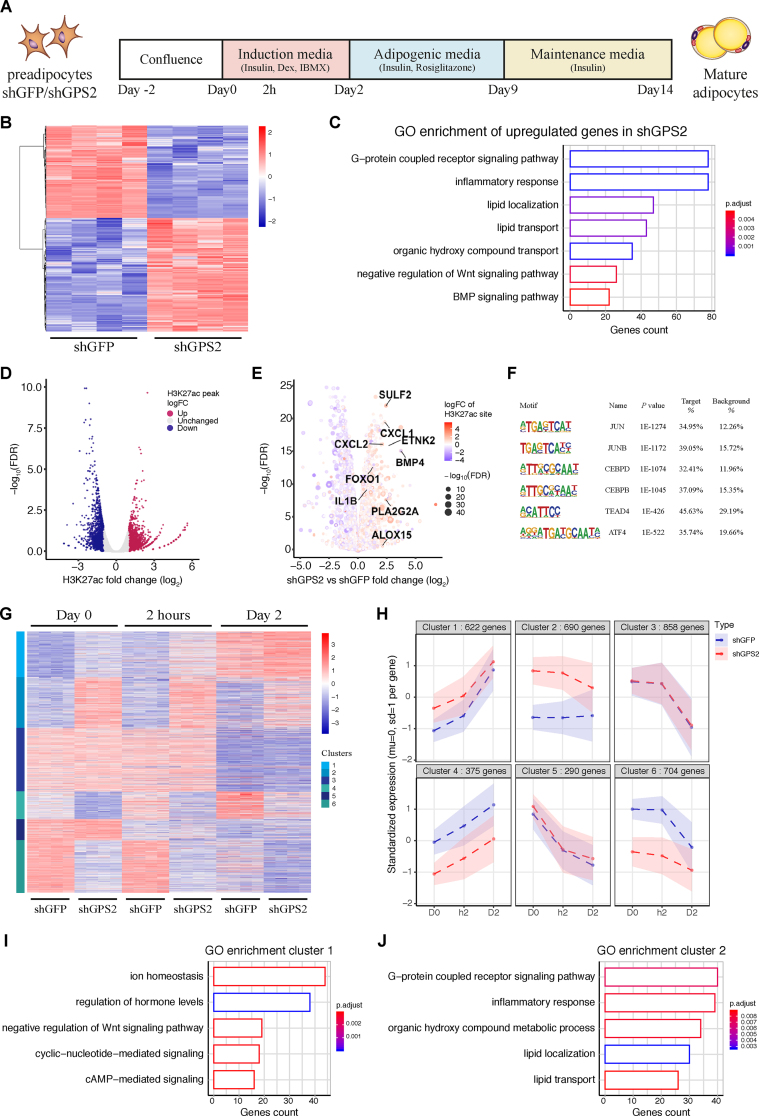
GPS2 depletion in preadipocytes increases transcription of genes involved in adipocyte commitment. (**A**) Schematic representation of the experimental design. hMADS shGFP/shGPS2 preadipocytes were 2 days at confluence before the induction of differentiation with a standard cocktail containing IBMX, dexamethasone, and insulin for 2 days. The cells were then maintained in adipogenic medium containing rosiglitazone until day 9, followed by a maintenance medium containing only insulin until day 14. (**B**) Heatmap of significant (adj. *p*-value < 0.05 and LogFC > 0.5 or LogFC < −0.5) gene expression changes in shGFP vs shGPS2 preadipocytes at day 0 (*n* = 4). (**C**) Gene ontology enrichment of significantly upregulated genes in shGPS2 preadipocytes. (**D**) Volcano plot showing the average logFC of H3K27ac peaks in shGPS2 vs shGFP preadipocytes (*n* = 2). (**E**) Volcano plot representing global mRNA expression along with logFC of H3K27ac (color from blue to red) and -log (FDR) (dot size) in shGPS2 vs shGFP preadipocytes. (**F**) Transcription factor motif analysis of GPS2-occupied regions in human preadipocytes. (**G**) Heatmap of significantly (adj. *p-*value < 0.05 and LogFC > 0.5 or LogFC < −0.5) changed gene expression in shGFP vs shGPS2 hMADS cells at day 0, 2 h, and day 2 (*n* = 4). (**H**) Cluster analysis of gene expression profiles during the early stage of adipocyte differentiation (days 0–2) in shGFP and shGPS2 cells (*n* = 4). (**I** and **J**) Gene ontology enrichment of clusters 1 and 2.

To identify the role of GPS2 during human adipocyte differentiation, we depleted GPS2 in hMADS preadipocytes using lentivirus shRNAs (Supplementary Figure 1A). GPS2-deficient hMADS cells displayed more than 80% reduction of GPS2 at the mRNA (Supplementary Figure 1B) and protein levels (Supplementary Figure 1C). Importantly, GPS2 depletion did not alter the mRNA levels of other HDAC3 corepressor complex subunits (Supplementary Figure 1D). After infection and selection, hMADS cells expressing shGFP or shGPS2 were induced to differentiate into mature adipocytes as illustrated in Figure 1A. We started to investigate the effect of GPS2 depletion in preadipocytes after two days of confluence, considered to be an undifferentiated status (day 0).

Transcriptome analysis via RNA-seq revealed that at this time point, 2,305 genes were differentially expressed between shGFP and shGPS2 cells (Figure 1B). Gene ontology (GO) analysis showed that the 1,271 upregulated genes were enriched for pathways related to G-protein coupled receptor signaling, in which the majority of the genes enriched in this pathway were genes also involved in inflammatory responses, which is the second most enriched pathway in shGPS2 cells. Moreover, the upregulated genes in the shGPS2 cells were enriched for pathways related to lipid metabolic processes, BMP signaling, and negative regulation of Wnt signaling (Figure 1C). In contrast, the 1,034 downregulated genes were enriched for different pathways, including those related to the differentiation of muscle or epithelial cells (Supplementary Figure 1E).

We further analyzed the epigenome changes in the hMADS cells via ChIP-seq for H3K27ac, a histone modification that marks active enhancers and promoters. We found that around 6000 promoter and enhancer loci had significantly increased or decreased H3K27ac levels in the shGPS2 compared to the shGFP cells (Figure 1D). Notably, the epigenome changes at regulatory regions correlated well with the transcriptome changes of the adjacent genes, that is, H3K27ac levels were higher in shGPS2-upregulated genes and lower in shGPS2-downregulated genes (Supplementary Figure 1F). In particular, among the highest upregulated genes with higher H3K27ac levels, we found *BMP4*, *IL1B*, and *CXCLs*, which are important for BMP signaling and inflammatory responses (Figure 1E), confirming the importance of GPS2 in controlling inflammatory processes in adipocytes [12].

Next, to define the chromatin occupancy of GPS2, referred to as cistrome, at day 0, we conducted ChIP-seq in regular hMADS cells. Transcription factor DNA-binding motif analysis revealed that binding sites for AP-1 members (c-JUN and JUNB) and C/EBPs were among the top motifs enriched at GPS-occupied chromatin regions. This suggests that these two transcription factor families, known to be involved in the regulation of inflammation and adipocyte differentiation, are potentially repressed by GPS2 in human preadipocytes (Figure 1F).

We then investigated the consequence of GPS2 depletion in adipose progenitor cells (hMADS) in response to the adipocyte differentiation cocktail composed of IBMX, dexamethasone, and insulin. We analyzed the transcriptomes at day 0 and 2 h post-induction to evaluate the responsiveness to adipogenesis and at day 2 of differentiation to characterize the induction phase before promoting adipogenesis through PPARγ activation (rosiglitazone treatment). We found that 3,539 genes were differentially expressed between shGFP and shGPS2 cells at least at one of these three time points (Figure 1G). We observed different transcriptome alterations at each time point and could further divide the genes into 6 different clusters. We found that the expression of the genes in clusters 1 and 2 was higher in the shGPS2 than in the shGFP cells at all three time points, while the expression of the genes in clusters 4 and 6 was always lower in the shGPS2 than in the shGFP cells (Figure 1H). In particular, we discovered that the genes in clusters 1 and 2 were linked to pathways important for the commitment to adipocytes, such as cAMP-mediated signaling or lipid localization and transport, as well as inflammatory response (Figure 1I–J). In contrast, genes in clusters 4 and 6 were linked to pathways that control epithelial differentiation and extracellular organization (Supplementary Figure. 1G–H). Moreover, we found that the expression of *BMP4*, *SULF2*, and *FOXO* in clusters 1 or 2 increased during the differentiation phase and this induction was higher in the shGPS2 cells than in the shGFP cells (Supplementary Figure 1I–K). The expression of the inflammatory genes *IL1B* and *CXCLs* decreased during induction of differentiation but was higher in the shGPS2 cells than in the shGFP cells (Supplementary Figure 1L–N). These results collectively indicate that in the absence of GPS2, there is a basal increased expression of genes involved in the commitment to adipocytes and a higher response to the induction phase of adipogenesis. Thus, GPS2 likely acts as corepressor at the early stages of adipogenesis.

### 3.2 GPS2 depletion in preadipocytes increases transcription of genes involved in lipid metabolism in mature adipocytes

**Figure 2:**
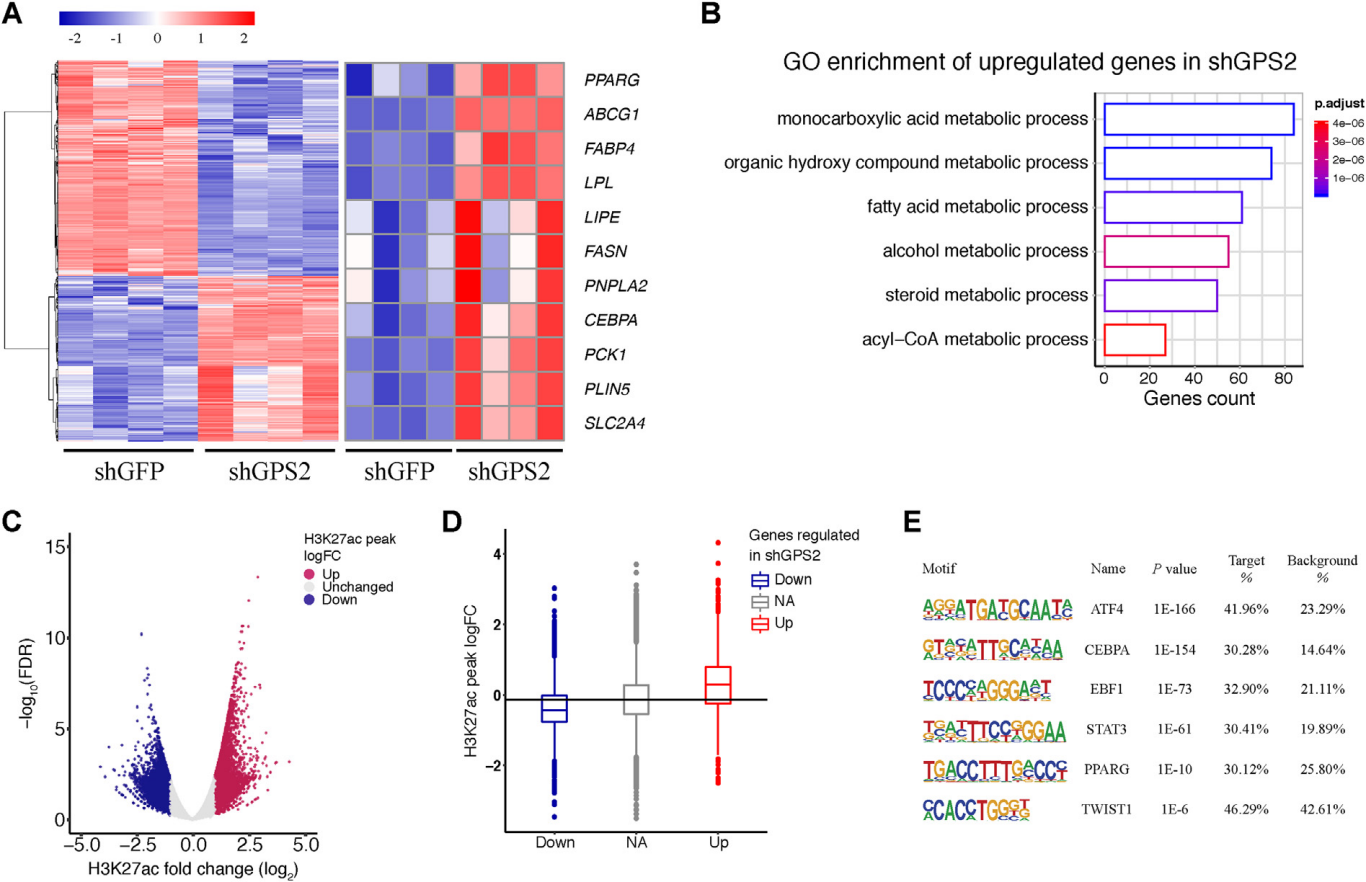
GPS2 depletion in preadipocytes increases transcription of genes involved in lipid metabolism in mature adipocytes. (**A**) Heatmap of significantly changed (adj. *p*-value < 0.05 and LogFC > 0.5 of LogFC < −0.5) gene expression in shGFP vs shGPS2 adipocytes at day 14 (left panel) and selection of adipocyte marker genes (right panel) (*n* = 4). (**B**) Gene ontology enrichment of significantly upregulated genes in shGPS2 adipocytes. (**C**) Volcano plot showing the average logFC of H3K27ac peaks in shGPS2 vs shGFP mature adipocytes (*n* = 2). (**D**) Box plot representing average logFC of co-localized H3K27ac peaks in downregulated, unregulated, and upregulated genes in shGPS2 cells at day 14. (**E**) Transcription factor motif analysis of GPS2-occupied regions in mature human adipocytes.

We further investigated the changes upon GPS2 depletion in fully differentiated adipocytes (day 14) characterized by adipocytes filled with lipid droplets. Transcriptome analysis revealed that more than 3,000 genes were significantly changed in GPS2-deficient adipocytes, including adipocyte marker genes encoding *PPARG*, *CEBPA*, and *FABP4* (Figure 2A). GO analysis showed that 1,349 upregulated genes were enriched for different metabolic processes such as organic hydroxy compounds, fatty acids, or steroid metabolic processes (Figure 2B). Next, an epigenome analysis revealed that 7,000 promoter and enhancer regions had significantly increased H3K27ac levels in the shGPS2 compared to the shGFP cells (Figure 2C). As in preadipocytes, the epigenome changes upon GPS2 depletion in differentiated adipocytes correlated well with the transcriptome changes, supporting a mechanism by which the loss of GPS2 triggers epigenetic activation of GPS2-sensitive loci (Figure 2D). Cistrome analysis revealed enrichment of C/EBPα, PPARγ, and TWIST1-binding sites, among others, at GPS2-occupied chromatin regions, suggesting that these transcription factors are potential targets of GPS2 in differentiated adipocytes (Figure 2E).

To confirm the genome-wide data at the single gene level and extend the analysis by including additional intermediate time points, we conducted kinetic profiling of mRNA expression using RT-qPCR throughout the entire adipocyte differentiation process (Supplementary Figure 2). The profiling in shGFP cells revealed that GPS2 was initially downregulated (at 6 h and day 2), but its expression was restored at day 14. GPS2 expression was low at all stages in the shGPS2 cells, confirming the efficacy of GPS2 depletion (Supplementary Figure 2A). We found a transient upregulation of the expression of *CEBPD* 2 h after induction until day 1 but no difference between the shGPS2 cells compared to the shGFP cells (Supplementary Figure 2B). The kinetic profile of *CEBPB* showed a strong increase 2 h post-induction and then decreased expression within 8 h post-induction, but expression again increased in the late stage of differentiation from day 9. Interestingly, in the early stage of differentiation, the expression of *CEBPB* was downregulated upon removal of GPS2, while it was upregulated in the late stage (Supplementary Figure 2C). In contrast, the kinetic profiles of *PPARG* and *CEBPA* showed a significant increase in mRNA expression during all time points of differentiation in the shGPS2 cells compared to the shGFP cells (Supplementary Figure 2D–E). Consistent with the upregulation of *PPARG* and *CEBPA* upon GPS2 depletion, the expression of key adipocyte marker genes controlled by these two transcription factors also increased at day 14 of differentiation in the shGPS2 cells compared to the shGFP cells (Supplementary Figure 2F). To further investigate the interactions of GPS2 with adipocyte transcription factors, we conducted co-immunoprecipitation assays (Supplementary Figure 2G). While C/EBPα or C/EBPβ did not interact with GPS2, interactions were observed with PPARγ along with C-JUN, PPARα, and SMRT, known interactors [22,32] serving as positive controls. Altogether, these data suggest that GPS2 acts as a corepressor of the adipogenic master transcription factor PPARγ, likely in cooperation with additional transcription factors, to control adipocyte differentiation and lipid metabolism in human adipocytes.

### 3.3 Loss of GPS2 induces adipocyte hypertrophy, phospholipid accumulation, and sphingolipid depletion

**Figure 3:**
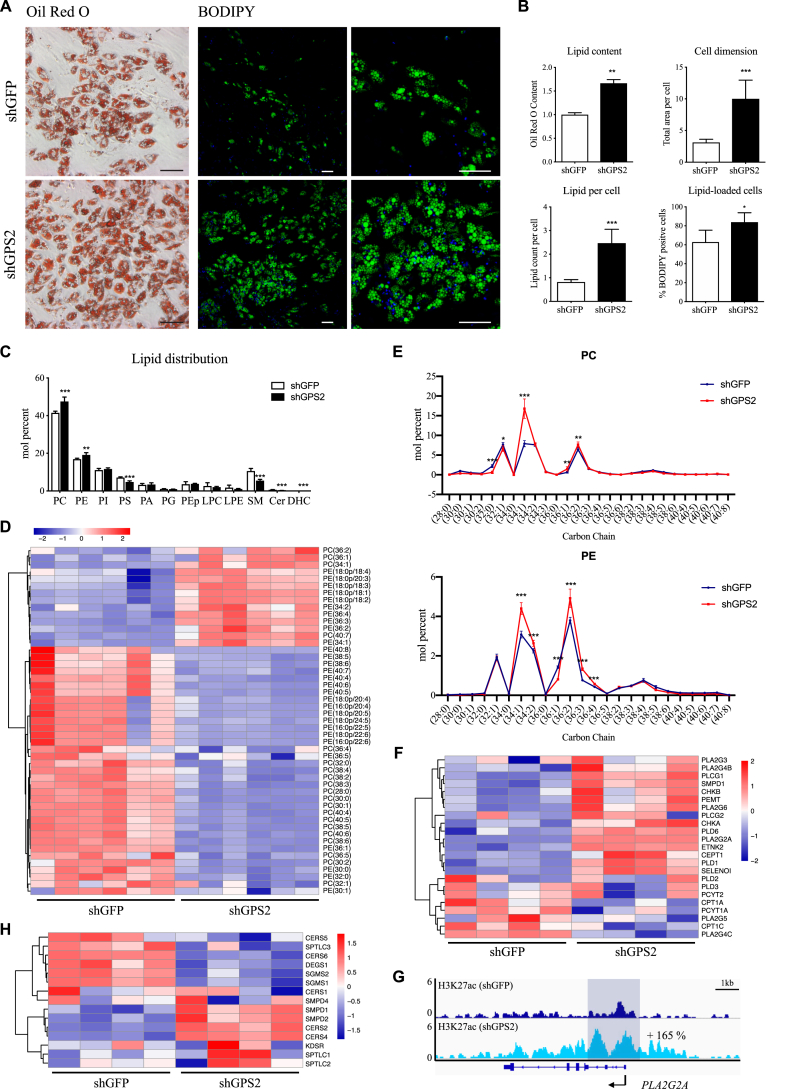
GPS2 depletion increases lipid accumulation, phospholipid accumulation, and sphingolipid depletion. (**A**) Representative images of Oil Red O staining and BODIPY immune-fluorescence staining conducted at day 14 in shGFP and shGPS2 cells. Scale bars, 100 μm. (**B**) Quantification of ORO staining (*n* = 3), quantification of cell dimension, lipid droplets per cell, and percentage of lipid-loaded cells (*n* = 5). (**C**) Distribution of lipid classes expressed as mol% of total phospho- and sphingolipids measured in shGFP and shGPS2 adipocytes (*n* = 6). (**D**) Heatmap of PC and PE species altered (FDR < 0.05) in shGFP and shGPS2 adipocytes (*n* = 6). (**E**) PC and PE species composition in shGFP and shGPS2 mature adipocytes (*n* = 6). (**F**) Heatmap of differentially expressed genes involved in PC and PE synthesis in shGFP vs shGPS2 adipocytes at day 14 (*n* = 4). (**G**) ChIP-seq tracks of H3K27ac at the *PLA2G2A* locus; the percentage of increase (shGPS2 vs shGFP) represents the area under the curve. (**H**) Heatmap of differentially expressed genes involved in Cer and SM synthesis in shGFP vs shGPS2 adipocytes at day 14 (*n* = 4). All of the data are represented as mean ± standard deviation (SD) (*n* = 3-5-6). ∗*p* < 0.05, ∗∗*p* < 0.01, ∗∗∗*p* < 0.001, and Student's t-test.

To investigate the phenotype of mature adipocytes after GPS2 depletion, we stained neutral lipids in the shGFP and shGPS2 adipocytes using Oil Red O and BODIPY (Figure 3A). Intriguingly, the loss of GPS2 during differentiation significantly increased triglyceride accumulation within adipocytes, adipocyte size (hypertrophy), the amount of lipid droplets per cell, and the percentage of differentiated cells without changing the lipid droplet dimensions (Figure 3B, Supplementary Figure 3A). To characterize the increased lipid accumulation, we analyzed the phosphosphingolipidome composition in the shGFP vs shGPS2 differentiated adipocytes. At the phospholipid class level, we found that the loss of GPS2 caused the depletion of most sphingolipid species including sphingomyelin (SM), ceramide (Cer), and dihydroceramide (DHC), whereas it enriched total phosphatidylcholine (PC) and phosphatidylethanolamine (PE) (Figure 3C). At the molecular level, the abundance of 149 phospholipid species (15 SM, 28 Cer, 9 DHC, 20 PC, 17 PE, 11 PI, 17 PS, 5 PA, 10 PG, 12 PEp, 3 LPC, and 2 LPE) were altered in adipocytes upon GPS2 deficiency (Supplementary Figure 3A) with an overall marked reduction of phospholipids containing polyunsaturated fatty acids (PUFA) (Figure 3D). It is noteworthy that shGPS2 adipocytes were enriched in monounsaturated PC and PE species and more specifically in oleic acid: 34:1 (mainly 16:0/18:1), 36:1 (mainly 18:0/18:1), 36:2 (mainly 18:1/18:1), and 36:3 (mainly 18:1/18:2) (Figure 3E). This phenotype of enrichment in the PC and PE lipids was further confirmed by analyzing the transcriptome and epigenome of phospholipid synthesis pathway genes (Supplementary Figure 3B). We found that GPS2 depletion increased the expression of genes encoding the major enzymes important for synthesizing PC and PE (Figure 3F). Interestingly, we found increased expression of 4 different PLA2 enzymes in the shGPS2 cells and increased H3K27ac at the promoters of some of these genes, for example, at *PLA2G2A* (Figure 3G). The transcriptome analysis of genes involved in sphingolipid metabolism (Supplementary Figure 3C) instead revealed decreased expression of major enzymes important for Cer biosynthesis, such as desaturase (DEGS1) or ceramide synthase (CERS5) and increased expression of sphingomyelinase (SMPD) (Figure 3H). Collectively, these results indicate that GPS2 depletion triggers adipocyte hypertrophy, sphingolipid depletion, and membrane remodeling with enrichment in phospholipids.

### 3.4 GPS2 regulates the expression of *ABCG1* and modulates LPL activity

The loss of sphingomyelin in adipocytes was recently reported to be modulated by ATP-binding cassette transporter ABCG1 [46]. Moreover, ABCG1 has been shown to play an important role in lipid homeostasis in mice [47] and humans [48]. In particular, two frequent single nucleotide polymorphisms (SNPs) in human *ABCG1* promoter have been associated with the activity of plasma lipoprotein lipase (LPL) and lipid accumulation. We also previously reported that GPS2 regulates ABCG1 expression through LXRs in human macrophages [34].

**Figure 4:**
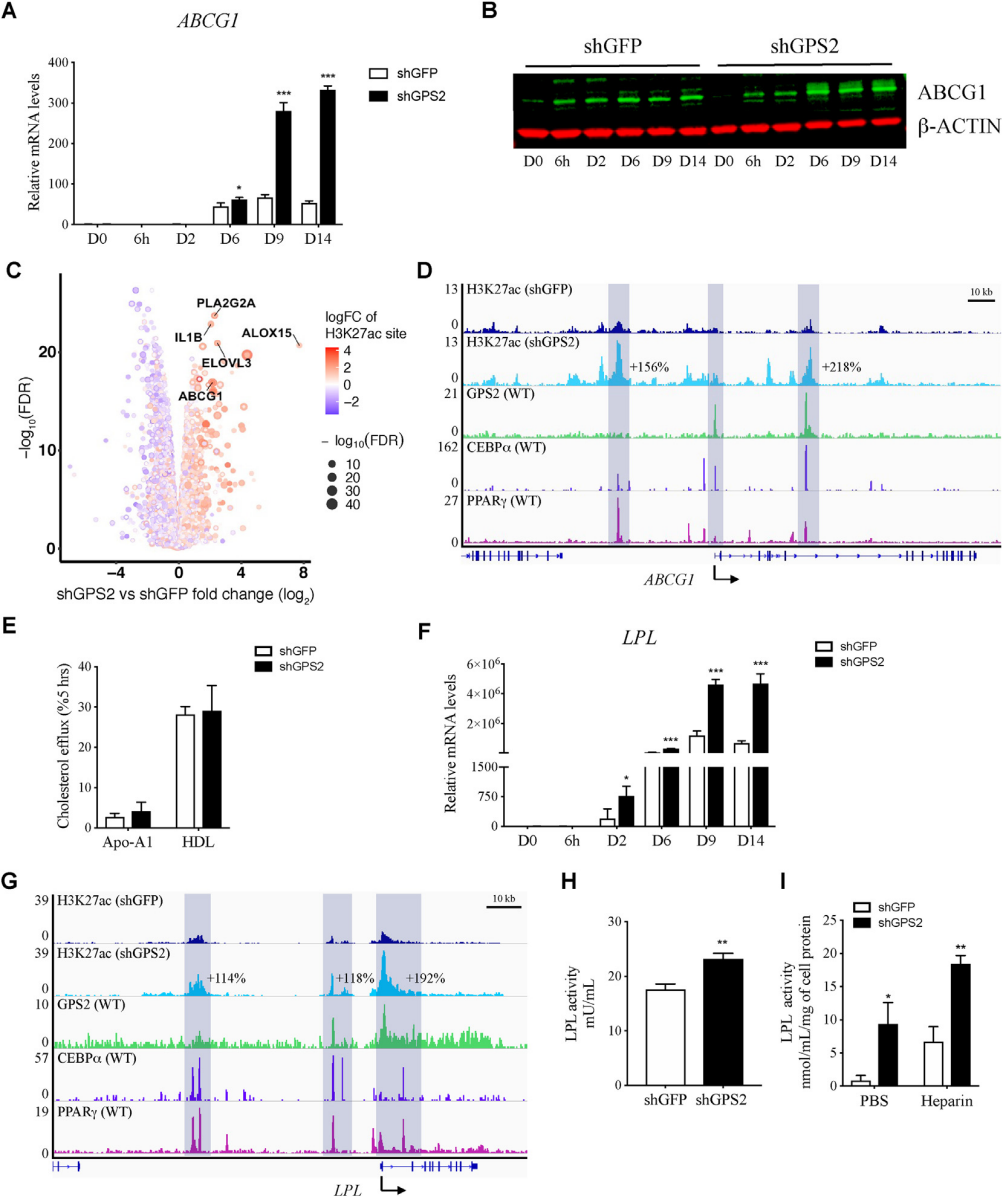
GPS2 depletion increases the expression of ABCG1 and expression and activity of LPL. (**A**) mRNA and (**B**) protein levels of ABCG1 during adipocyte differentiation in shGFP and shGPS2 hMADS cells (*n* = 3). (**C**) Volcano plot of global gene expression along with logFC of H3K27ac sites (color from blue to red) and -log (FDR) (dot size) in shGPS2 vs shGFP adipocytes at day 14. (**D**) ChIP-seq tracks of H3K27ac, GPS2, C/EBPα (GSE24326), and PPARγ (P5_adipocytes GSE115421) at the *ABCG1* locus in human adipocytes. (**E**) Cholesterol efflux to apoA-1 or HDL determined in shGFP and shGPS2 adipocytes, day 14 (*n* = 5). (**F**) *LPL* mRNA levels in shGFP vs shGPS2 adipocytes, day 14 (*n* = 3). (**G**) ChIP-seq tracks of H3K27ac, GPS2, C/EBPα (GSE24326), and PPARγ (P5_adipocytes GSE115421) at the *LPL* locus in human adipocytes. (**H**) Intracellular and (**I**) secreted LPL activity in shGFP vs shGPS2 adipocytes, day 14 (*n* = 3). All of the data are represented as mean ± standard deviation (SD). ∗*p* < 0.05, ∗∗*p* < 0.01, ∗∗∗*p* < 0.001, and Student's t-test.

Based on these implications, we investigated the effect of the loss of GPS2 on the expression and activity of ABCG1 in hMADS cells. Starting from day 6 of differentiation, the differentiation kinetic showed a significant increase in ABCG1 mRNA and protein levels in the shGPS2 cells compared to the shGFP cells (Figure 4A–B). Consistent with these RT-qPCR data, RNA-seq identified *ABCG1* as one of the most upregulated genes in GPS2-depleted adipocytes, and this was further corroborated by an increase in H3K27ac levels at the *ABCG1* promoter and enhancer regions (Figure 4C). Interestingly, a comparison between different ChIP-seq profiles at the *ABCG1* locus in human adipocytes revealed that GPS2, C/EBPα, and PPARγ co-occupy some of these regulatory regions with altered H3K27ac (Figure 4D). Given the well-established role of ABCG1 in cholesterol efflux in non-adipocyte cell types, we determined free cholesterol efflux mediated by Apo-A1 or HDL in shGFP vs shGPS2 adipocytes but did not find significant differences (Figure 4E).

To investigate the role of GPS2 in mature human adipocytes, we depleted GPS2 in pre-differentiated hMADS cells (day 9) using lentivirus shRNA. GPS2-deficient adipocytes displayed an approximate 60% reduction in *GPS2* at the mRNA level (Supplementary Figure 4A). Interestingly, while the expression of *PPARG* and *CEBPA* did not change upon partial GPS2 depletion (Supplementary Figure 4B), *ABCG1* expression slightly but significantly increased (Supplementary Figure 4C).

To further explore the mechanisms by which GPS2 depletion increases triglyceride accumulation, we investigated the effect of GPS2 depletion on LPL transcription, followed by determining LPL activity, which has been proposed to be modulated by the presence of sphingomyelin in the plasma membrane [49]. We found a significant increase in *LPL* mRNA in the shGPS2 cells compared to the shGFP cells starting from day 2 of differentiation (Figure 4F). Interestingly, we found that upon depletion of GPS2, the levels of H3K27ac in the promoter and enhancer of the *LPL* locus increased, and these altered regulatory regions were co-occupied by GPS2, C/EBPα, and PPARγ (Figure 4G). Consistent with the mRNA changes, we observed an increase in intracellular LPL activity (Figure 4H) and an increase in extracellular LPL activity at basal or upon heparin stimulation (Figure 4I) in GPS2-depleted adipocytes.

In summary, these data suggest that GPS2 controls *ABCG1* expression, likely interfering with multiple steps in the transcriptional activation process, including the repression of PPARγ and/or C/EBPα activity and the antagonism of H3K27 acetylation at enhancers and promoters. Thereby, the loss of GPS2 in adipocytes triggers a series of alterations involving upregulation of ABCG1, depletion of sphingomyelin from the plasma membrane, and increase in LPL activity and triglyceride storage.

### 3.5 GPS2 expression correlates with type 2 diabetes and lipid management in adipose tissue of obese patients

**Figure 5:**
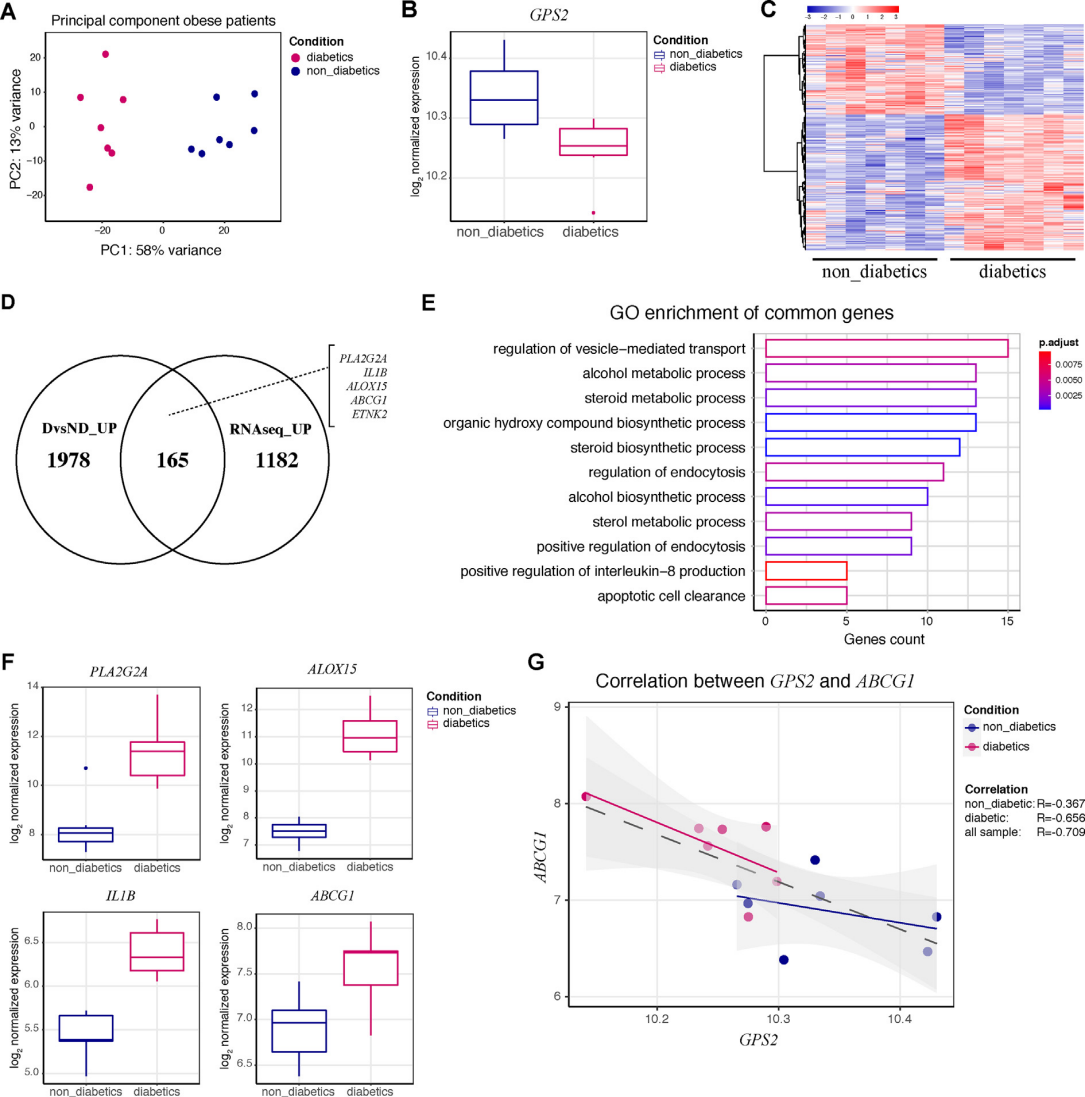
Correlations of *GPS2* expression with *ABCG1* expression, diabetic status, and lipid management in omental adipose tissue of obese patients. (**A**) Principal component (PC) analysis of transcriptome data from obese patients grouped by type 2 diabetic status (*n* = 7 obese non-diabetic and *n* = 7 obese diabetic). (**B**) Box plot representing log_2_ normalized GPS2 expression in non-diabetic and type 2 diabetic obese patients (*n* = 7 obese non-diabetic and *n* = 7 obese diabetic). (**C**) Heatmap of significantly changed ( *p*-value < 0.05 and LogFC > 0.5 or LogFC < −0.5) gene expression in non-diabetic vs diabetic obese patients (*n* = 7 obese non-diabetic and *n* = 7 obese diabetic). (**D**) Venn diagram representing the commonly upregulated genes (diabetic vs non-diabetic patients) and hMADS cells (shGPS2 vs shGFP). (**E**) Gene ontology enrichment of the 165 commonly upregulated genes shown in (**D**). (**F**) Box plot representing log_2_ normalized expression of *PLA2G2A*, *ALOX15*, *IL1B*, and *ABCG1* in non-diabetic vs diabetic obese patients (*n* = 7 non-diabetic and *n* = 7 diabetic). (**G**) Correlation analysis of *GPS2* with *ABCG1* mRNA levels in human obese omental adipose tissue (*n* = 7 non-diabetic and *n* = 7 diabetic).

To determine the clinical relevance of our findings in human disease, we analyzed the transcriptome by RNA sequencing omental adipose tissue from 14 obese patients with or without type 2 diabetes (Table S1). We observed that the two groups, non-diabetic and diabetic, exhibited clear differences in their gene expression patterns (Figure 5A). As previously reported by our group [36], we found that the mRNA level of GPS2 was lower in the obese diabetics than obese non-diabetics (Figure 5B). The genome-wide comparison of the transcriptomes between the non-diabetics and diabetics revealed that more than 3,500 genes were significantly regulated by the presence of diabetes in the obese patients (Figure 5C). Therefore, we compared the upregulated genes in the diabetic vs non-diabetic patients with upregulated genes in the shGPS2 vs shGFP hMADS cells. We found that 165 genes overlapped in these two conditions (Figure 5D). GO analysis of the common genes showed that they belonged to inflammatory pathways as well as pathways of lipid synthesis and metabolism (Figure 5E). Moreover, we found that the expression of the highest upregulated genes in GPS2-depleted adipocytes, such as *PLA2G2A*, *ALOX15*, *IL1B*, and *ABCG1*, increased in the diabetic condition compared to the non-diabetic (Figure 5F). Furthermore, we found a significant inverse correlation between *GPS2* and *ABCG1* expression independent of diabetic status (Figure 5G). We conclude from these data that GPS2 expression and function can correlate with the diabetic status in obese patients, and that GPS2 potentially regulates the expression of metabolic genes including *ABCG1* involved in triglyceride storage and lipid remodeling in human adipocytes.

## 4 Discussion

**Figure 6:**
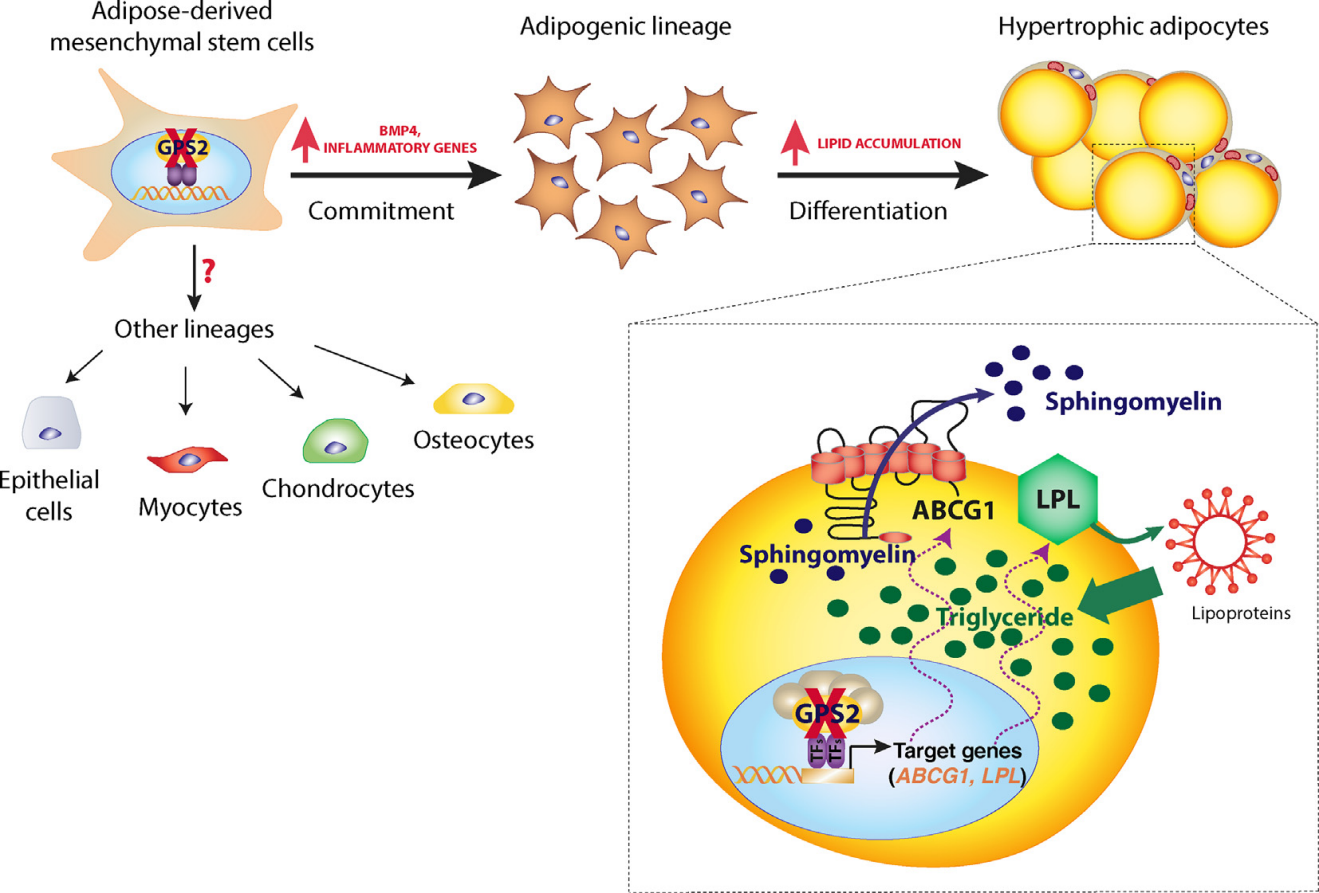
Model highlighting the identified dual role of GPS2 during human adipocyte differentiation. In the early stage of adipogenesis, loss of GPS2 triggers the commitment of fibroblast-like progenitors toward the adipogenic lineage by the responses to the adipogenic cocktail and the expression of inflammatory and metabolic genes. In the late stage of adipogenesis, the loss of GPS2 results in adipocyte hypertrophy with increased triglyceride (TG) accumulation. Consistent with this phenotype, the loss of GPS2 increases transcription and activity of lipoprotein lipase (LPL) that acts on circulating lipoproteins to promote intracellular TG accumulation. Additionally, the loss of GPS2 increases *ABCG1* expression that contributes to the depletion of intracellular sphingomyelin (SM) via increased efflux from adipocytes, further contributing to adipocyte hypertrophy.

In this study, we identify a previously unknown role of the corepressor complex subunit GPS2 in the regulation of pathways governing the differentiation and hypertrophy of human adipocytes. We demonstrate that depletion of GPS2 induces upregulation of multiple genes involved in adipogenesis and lipid metabolism that is associated with a deep remodeling of the adipocyte lipidome, such as the accumulation of triglycerides, phosphatidylcholine, and phosphatidylethanolamine and depletion of sphingolipids. We show that the loss of GPS2 in preadipocytes causes increased *ABCG1* and *LPL* gene expression, likely via de-repression of their promoter and enhancers and increase in LPL activity, two crucial elements for triglyceride accumulation linked to hypertrophy (for a model, see Figure 6). The hMADS cell-derived findings were validated by gene expression analysis of an obese cohort, where *GPS2* was downregulated in diabetic patients and negatively correlated with the expression of *ABCG1*.

Our findings are consistent with the earlier discovered peculiar role of ABCG1 in adipocytes and provide novel insights into the transcriptional regulation of *ABCG1* expression in human adipocytes. Beyond the well-described role of ABCG1 in cholesterol efflux from macrophages, these earlier studies suggested a critical role of ABCG1 in the regulation of adiposity. *Abcg1* knockout mice were protected against high-fat diets and had improved glucose tolerance and reduced adipose tissue mass with a significant reduction in adipocyte size [50]. Moreover, it has been shown that ABCG1 mediates sphingomyelin efflux, which contributes to increased LPL activity and consequently triglyceride storage in adipocytes [46]. Interestingly, we also found increased expression of *SMPD1*, a sphingomyelinase that catalyzes the hydrolysis of sphingomyelin, which may contribute to sphingomyelin depletion in shGPS2 cells. However, it is well established that SMPD1 is important for determining the ceramide pool [51]. As ceramides are reduced in a similar way to sphingomyelin in shGPS2 cells, we believe that the reduction of sphingomyelins is not the result of the increased expression of SMPD1 but could be the result of increased efflux mediated by ABCG1. Moreover, our data extend current knowledge about the role of ABCG1 in adipocytes by integrating GPS2 as a coregulator of its expression. Interestingly, the GPS2-occupied *ABCG1* promoter region contains SNP rs1378577, which was associated with altered LPL activity in a dyslipidemic population in the Regression Growth Evaluation Statin Study [48]. This SNP promoter can potentially affect the chromatin binding of GPS2, along with target transcription factors such as PPARγ or C/EBPα, and consequently alter *ABCG1* expression in adipocytes.

The GPS2-ABCG1 pathway emphasizes that coregulator-transcription factor networks are often cell type-selective. We demonstrate here that GPS2 cooperates with a specific transcription factor network, including PPARγ, to control *ABCG1* expression in human adipocytes, and we demonstrated previously that GPS2 cooperates with a different transcription factor network, including the oxysterol receptor LXR, to control *ABCG1* expression in human macrophages [34]. These studies emphasize that the physiological roles of the respective GPS2-ABCG1 pathways and the underlying mechanisms are different in adipocytes and macrophages. Specifically, cholesterol efflux seems to be regulated only by GPS2-ABCG1 in macrophages but not in adipocytes. Conversely, whether sphingomyelin efflux is regulated in macrophages as in adipocytes remains an interesting open issue to address in the future.

Our study expands the knowledge about the activity control of PPARγ and C/EBPs, both master regulators of adipogenesis and adipocyte metabolism in mice and humans. GPS2 controls these transcription factors presumably via two interconnected mechanisms. First, GPS2 depletion in preadipocytes increased the mRNA expression of both *PPARG* and *CEBP*s and this mechanism is possibly linked to the second, for example, via an autoregulatory loop or a third unknown factor regulated by GPS2. Second, GPS2 depletion caused increased H3K27ac at co-occupied promoters and enhancers, indicative of epigenetic remodeling linked to the activation of the transcription factors along with their target genes, thereby triggering pathways governing adipogenesis and lipid metabolism. Considering that the composition and activity of transcription factor networks are dynamic and change during adipogenesis, GPS2 likely acts as corepressor for different sets of transcription factors at the co-occupied regulatory regions in preadipocytes vs mature adipocytes. Specifically, our current study does not directly address which transcription factors are the targets of GPS2 repression in preadipocytes that express relatively low levels of PPARγ before induction. However, the ChIP-seq motif analysis in preadipocytes revealed several candidates that remain to be investigated. Interestingly, studies using mouse [52] and human adipocytes [53] suggests that adipogenesis is driven by the activation of a highly connected network of cooperating transcription factors, while repression of distinct transcription factor networks acts as a molecular brake on adipogenesis [53]. Our GPS2 data presented herein, along with previous data on SMRT in 3T3-L1 cells [26], suggest that some of these repressing transcription factors, including C/EBP and AP-1 family members, could be targets for GPS2 (and the GPS2-SMRT corepressor complex) at early stages of human adipogenesis.

There seems to be a discrepancy of our study with previous studies showing that the loss of GPS2 decreases PPARγ expression and adipogenesis [31] and that GPS2, at some PPARγ target genes (lipolysis), acts as a coactivator in 3T3L1 cells [33]. The most likely reason is that the regulatory networks are different between mice and humans. This includes the limited or lacking conservation of transcription factor binding sites and enhancers, differences in transcription factor and coregulator levels, and post-transcriptional modifications (including PPARγ phosphorylation), thus affecting the ratios and interaction dynamics in human vs mouse adipocytes. However, one study [54] reported that shRNA depletion of GPS2 in 3T3-L1 cells results in increased adipogenesis and increased lipid storage, just as we observed in hMADS cells. Another 3T3-L1 study [26] suggested that the depletion of SMRT has similar consequences to the depletion of GPS2, supporting our earlier data in which the genomic GPS2 action in human adipocytes requires cooperation with SMRT [12]. Regarding the coactivator role of GPS2 [33], we have no mechanistic explanation to offer other than suggesting the aforementioned mouse-human differences. Our current hMADS data suggest that this coactivator mechanism does not operate in regulating *ABCG1* or lipolysis genes that are upregulated upon GPS2 depletion. Some downregulated genes may be potentially regulated this way, but we have not yet explored the regulation of these genes.

Several studies suggested that adipocyte size is an important predictive marker and a predisposing factor of adipose tissue dysfunction in obese and non-obese individuals [55]. Hypertrophy is associated with an increased risk of developing type 2 diabetes independent of obese status [56]. Normal-weight subjects with a genetic predisposition for type 2 diabetes show improper adipose cell enlargement and develop insulin resistance before obesity [57]. Adipose-derived LPL, the rate-limiting enzyme for triglyceride storage in adipocytes, has been reported to be an important factor influencing adipocyte hypertrophy [58]. In accordance with this evidence, depletion of GPS2 induced significantly increased activity of LPL accompanied by a marked accumulation of triglycerides. A variety of studies also support that sphingomyelins strongly inhibit LPL activity in lipoprotein metabolism [49,59], strengthening our hypothesis that the depletion of sphingomyelins contributes to increased LPL activity in GPS2-depleted hMADS cells.

Studies in mice and humans suggest that adipocyte expansion proceeds until a critical cell volume is reached and that hypertrophic adipocytes further increase adipocyte numbers [60]. Under appropriate stimulation, progenitor cells residing in the stromal vascular fraction of adipose tissue become committed to the adipocyte lineage, giving rise to adipocyte precursors. Interestingly, we discovered that removal of GPS2 resulted in increased expression of BMP4, an important trigger for the commitment of multipotent stem cells to the adipocyte lineage [61], in particular in response to cAMP signaling, but also associated with adipocyte size and insulin sensitivity [62]. Based on this, we propose that GPS2 plays a dual role during adipogenesis. In the early stage of adipogenesis, GPS2 regulates the commitment to adipocytes because if GPS2 is removed, more cells are pushed to become adipocytes and cells are more prone to accumulate lipids. In the late stage of adipogenesis, GPS2 regulates sphingolipid metabolism via ABCG1 and presumably plays a pivotal role in modulating the hypertrophic phenotype of mature adipocytes. Thus, the loss or inhibition of GPS2 could be beneficial in preadipocytes to promote recruitment and differentiation of new fat cells, but detrimental in mature adipocytes, leading to adipocyte hypertrophy and a pro-inflammatory profile.

Clinical evidence suggests that obesity and insulin resistance are characterized as chronic inflammatory conditions that are likely initiated in the adipose tissue via pro-inflammatory activation and macrophage infiltration [63]. In our study, we observed an increase in the expression of inflammatory genes upon removal of GPS2, consistent with earlier findings in adipose tissue and macrophages [12,31,32]. These studies also revealed that GPS2 expression is downregulated in adipocytes in obese humans, thereby contributing to the transcriptional and epigenetic remodeling at inflammatory promoters including *IL-6*, *IL-8*, and *CCL2/MCP-1*, favoring inflammation of adipose tissue in obese subjects [12]. In this study, we propose that the expression of *GPS2* is regulated by the transcription factor TWIST1. Interestingly, other studies reported the function of TWIST1 during white adipose tissue inflammation and its correlation with obesity and insulin resistance [64,65]. Of note, inflammation has been reported to increase adipogenesis, contributing to healthy adipose tissue remodeling and expansion [66]. Therefore, the increased inflammation observed herein in the GPS2-depleted hMADS cells might be a contributing factor during the early stage of adipogenesis by committing more cells to the adipocyte lineage. Further, it has been demonstrated that inflammation can be influenced by the PUFA content in the phospholipid composition. In particular, the ratio of saturated fatty acid (SFA)/PUFA protects against inflammation and insulin resistance [67,68]. Therefore, the reduction in PUFAs could be another contributing factor to increased inflammation observed upon GPS2 deficiency.

The human relevance of our *in vitro* study is supported by the transcriptome analysis of obese subjects who are non-diabetic or type 2 diabetic. Our findings thereby strengthen the involvement of GPS2 in regulating transcriptional cascades in human adipocytes in response to metabolic stress that accelerates the development of type 2 diabetes. This enables us to propose that GPS2 acts as an epigenetic modulator of gene expression alterations that contribute to the diabetic phenotype. It has been shown that, apart from the genetic component, the individual epigenetic signature contributes to the development of obesity and type 2 diabetes [69,70]. In light of the identified GPS2-ABCG1 pathway, it is of interest that DNA methylation at the *ABCG1* locus is associated with an increased risk of developing type 2 diabetes [71]. Also, adipose tissue DNA methylation is altered in diabetic subjects and this may have an impact on inflammation and lipid metabolism [72]. Although it is unknown whether these DNA-methylations are mechanistically linked to epigenetic histone modifications such as the H3K27ac enhancer marker, our hMADS cell data allow us to speculate that type 2 diabetes-associated GPS2 alterations trigger a massive epigenetic remodeling of this histone marker in human adipocytes in the context of type 2 diabetes, including at the *ABCG1* locus.

## Author contributions

S.B. conceived the study, with major input from N.V. and E.T. S.B. conducted most of the experiments. S.B., N.L., and E.M. analyzed the NGS data. E-Z.A. provided the hMADS cells. W.L.G. performed and analyzed the lipidome profile. R.B., S.L., A.S., J.-F.G., and N.V. collected and analyzed the human samples. S.B. wrote the manuscript. E.T. further edited the manuscript with input from all of the co-authors.
