# Supplementary material for: Loss of G protein pathway suppressor 2 in human adipocytes triggers lipid remodeling by upregulating ATP binding cassette subfamily G member 1
Source: Mol Metab. 2020 Aug 13;42:101066. doi: 10.1016/j.molmet.2020.101066 (PMC7509237; doi:10.1016/j.molmet.2020.101066)
Supplement: Multimedia component 1 [file mmc1.pdf]

Loss of G protein pathway suppressor 2 in human adipocytes triggers lipid remodeling through upregulation of ATP binding cassette subfamily G member 1

Barilla et al.

Supplementary data

Supplementary Figure 1

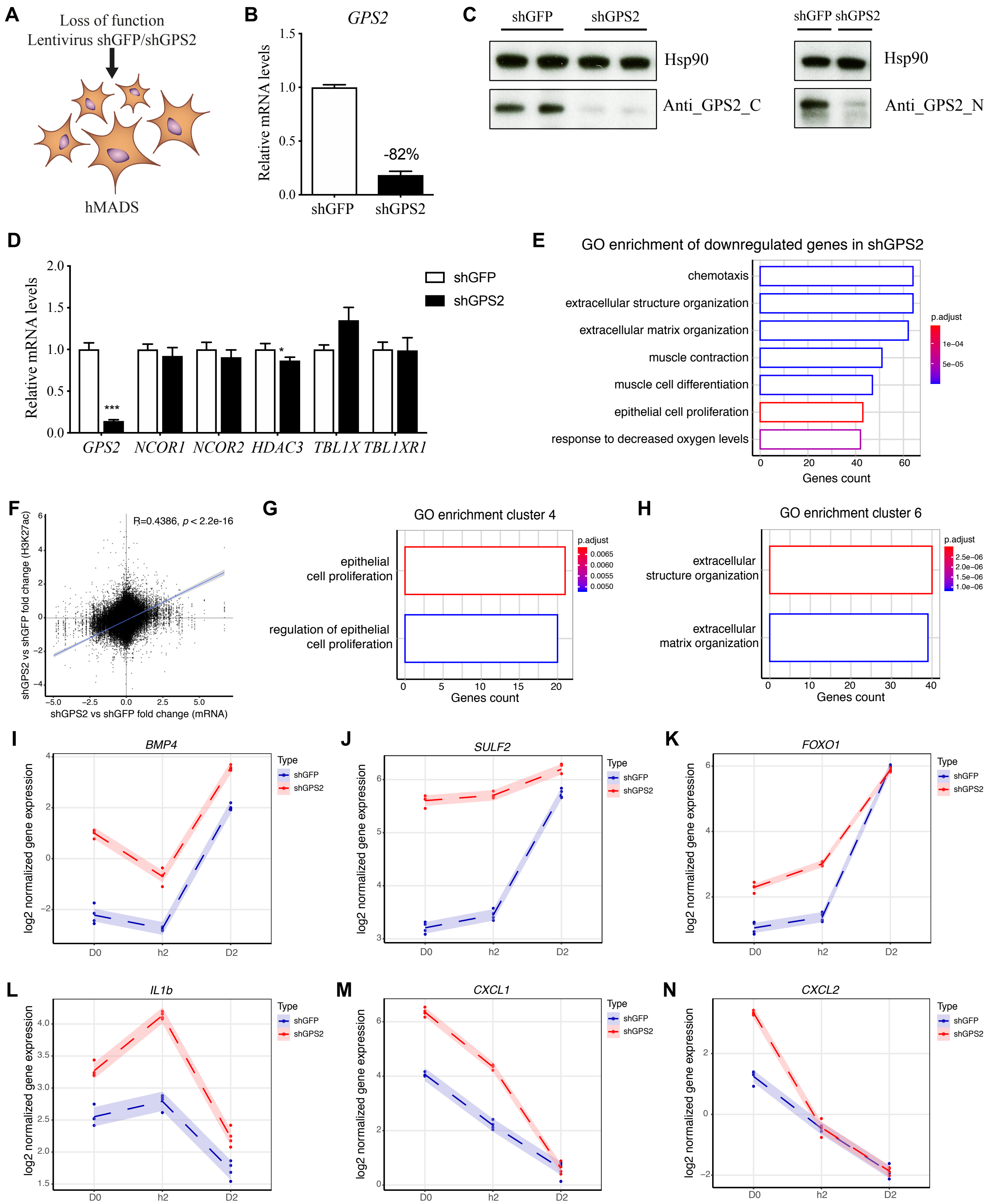

Supplementary Figure 1. Generation and validation of GPS2-depleted hMADS cells. (**A**) Schematic representation of hMADS transduced with lentivirus expressing shRNA targeting GFP (negative control) or shRNA targeting human GPS2. (**B**) mRNA level of GPS2 in shGFP and shGPS2 cells. (**C**) Western blot determination of GPS2 protein levels in shGFP and shGPS2 cells using two different antibodies recognizing either the GPS2 C-terminus (left) or the N-terminus (right). (**D**) RT-qPCR analysis of mRNA expression of all core subunits of the HDAC3 corepressor complex in shGFP and shGPS2 cells (n=3). (**E**) Gene ontology enrichment analysis of significantly downregulated genes in shGFP versus shGPS2 pre-adipocytes (day 0). (**F**) Global correlation analysis of mRNA logFC with H3K27ac site logFC in shGPS2 versus shGFP cells, nonparametric Spearman's test. (**G**,**H**) Gene ontology enrichment analysis of gene clusters 4 and 6. (**I**-**N**) Expression profile of representative genes from different cluster in (Figure 1H). All data are represented as mean ± standard deviation (SD). *$p<0.05$, ***$p<0.001$, Student's t-test.

Supplementary Figure 2

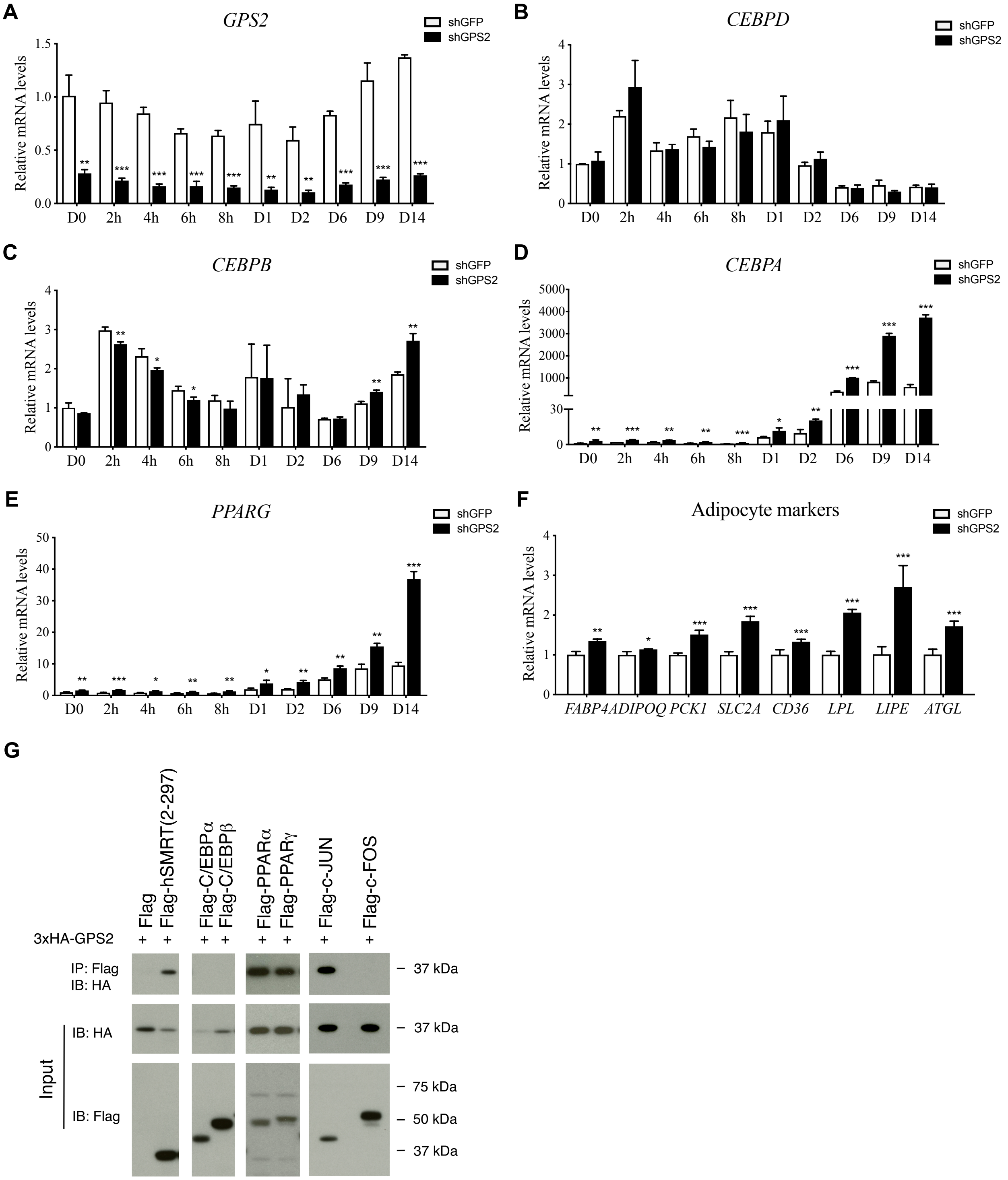

Supplementary Figure 2. GPS2 depletion leads to increased expression of adipogenic transcription factors *PPARG* and *CEBPA* and their target genes. (**A**-**E**) mRNA levels of *GPS2*, *CEBPD*, *CEBPB*, *CEBPA* and *PPARG* during adipocyte differentiation in shGFP and shGPS2 hMADS cells. (**F**) mRNA level of adipocyte marker genes at day 14 in shGFP and shGPS2 hMADS cells. (**G**) Co-immunoprecipitation of HA-GPS2 with Flag hs-SMRT, PU.1, CEBPs, PPARs, JUN and FOS in HEK293 cells. All data are represented as mean ± standard deviation (SD). *p<0.05, **p<0.01, ***p<0.001, Student's t-test.

Supplementary Figure 3

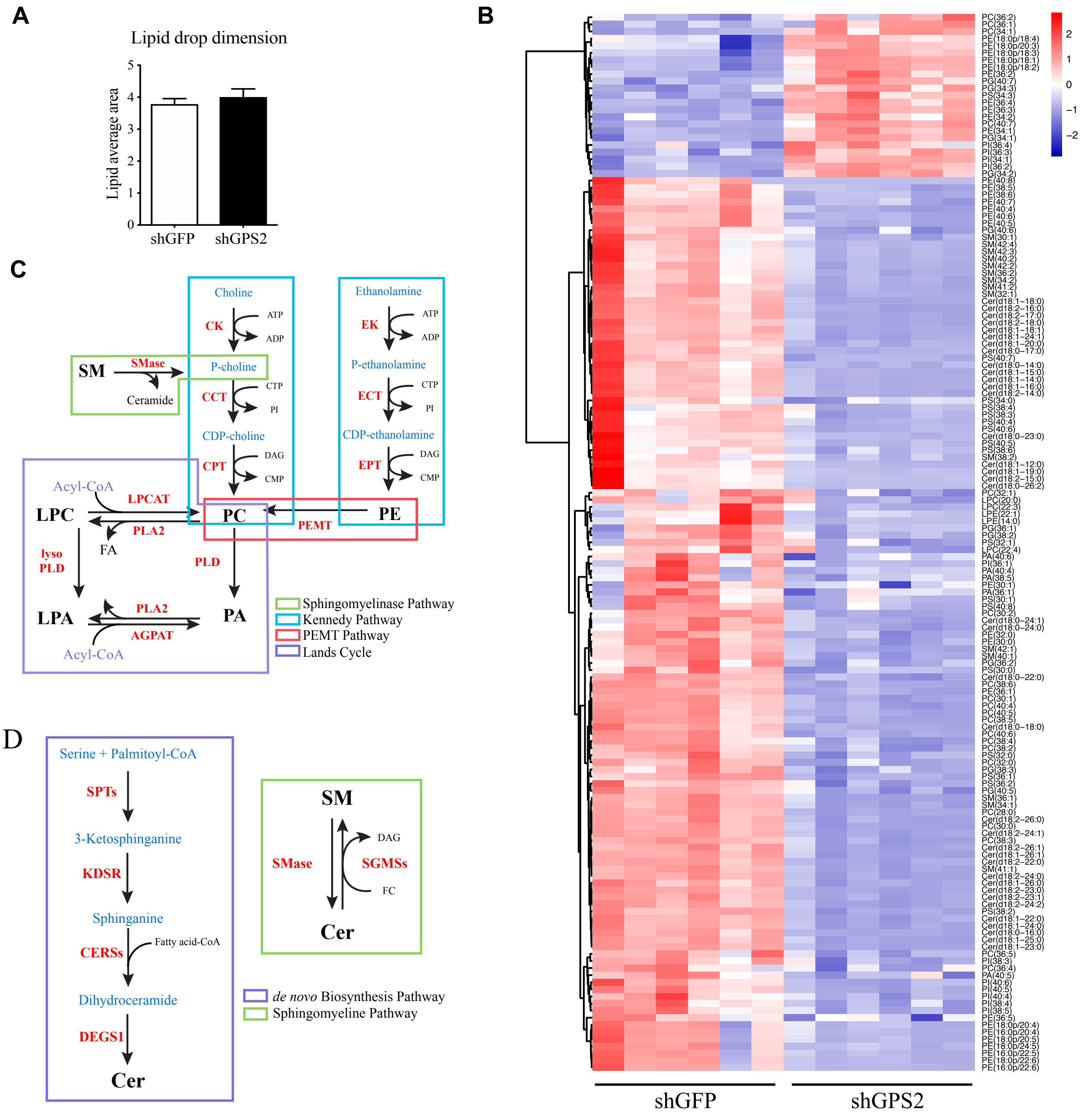

Supplementary Figure 3. Regulation of phospholipid pathways. (**A**) Quantification of lipid droplet dimension in shGFP and shGPS2 cells at day 14 (n=5). (**B**) Heatmap of top phospholipid species altered (FDR<0.05) in shGFP and shGPS2 adipocytes (n=6). (**C**) Phospholipid synthesis pathways with enzymes involved in red. (**D**) Ceramide and sphingomyelin synthesis pathways with enzymes involved in red.

Supplementary Figure 4

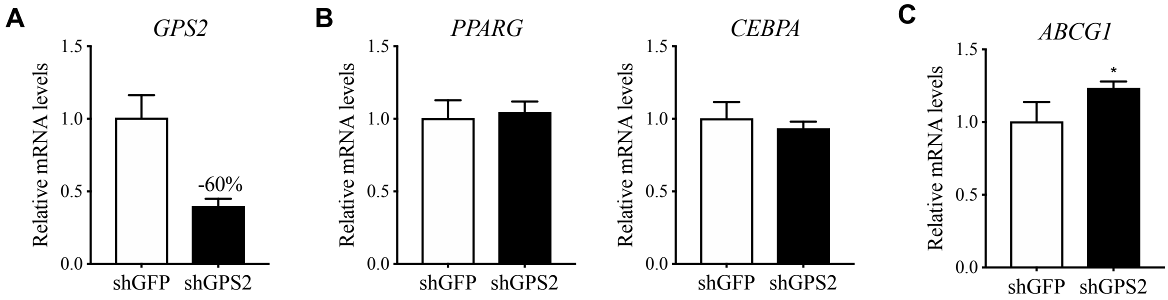

Supplementary Figure 4. GPS2 knockdown in mature adipocytes. (**A**) mRNA level of GPS2 in shGFP and shGPS2 hMADS cells. (**B**) mRNA levels of *PPARG* and *CEBPA* in shGFP and shGPS2 hMADS cells. (**C**) mRNA level of *ABCG1* in shGPS and shGPS2 hMADS cells. All data are represented as mean ± standard deviation (SD). *p<0.05, Student's t-test.

Supplementary Table 1 – Clinical and anthropometric variables in human study population

	Obese non-diabetic	Obese diabetic	*p*
N	7	7	
Sex	F	F	
Age (years)	36 ± 12	39 ± 10	0.68
BMI (kg/m^2)	41 ± 4	42 ± 3	0.67
Total cholesterol (g/L)	1.96 ± 0.23	1.70 ± 0.38	0.24
HDL (g/L)	0.58 ± 0.16	0.48 ± 0.12	0.35
LDL (g/L)	1.18 ± 0.29	1.02 ± 0.33	0.40
HbA1c (%)	5.26 ± 0.45	6.92 ± 0.15	< 0.0001
CRP (mg/L)	4.13 ± 1.94	5.74 ± 1.79	0.19

Mean ± SD

Supplementary Table 2 – Primers for RT-qPCR

Gene	Forward (5'-3')	Reverse (5'-3')
RPLP0	GTGTTCGACAATGGC	GACACCCTCCAGGAA
GPS2	GGGGAGCCCTGGAGGACACA	TGAGGTGGGGGCTGAGCAGT
NCOR1 (NCoR)	TTCAGCGAGTTGGGCAGAGGG	GGGTCCTCCATAAGCCCATTCATGT
NCOR2 (SMRT)	ATGGACCGCGTGGACCGAGA	GCAGCTTCAGCCTTCTTCCGGTT
HDAC3	GCCCCATCGCCTGGCATTGA	ACACTGGGCAGTCATCGCCT
TBL1X (TBL1)	CAACGAGGATGGCACAGTGTTCG	AAACGCCGGCTGAGGTCGTG
TBL1XR1 (TBLR1)	GCAAATGGGGAGGAGAATGGAGCA	TGCTGTTGAGTCTCCAGACCCTG
CEBPD	GGAGAGACTCAGCAACGACC	TTGCGCTCCTATGTCCCAAG
CEBPB	AACCAACCGCACATGCAGAT	GGCAGAGGGAGAAGCAGAGAGT
PPARG	CATAATGCCATCAGGTTTGGGCGG	CGCCCTCGCCTTTGCTTTGG
CEBPA	CTTGTGCCTTGGAAATGCAA	GCTGTAGCCTCGGGAAGGA
FABP4	TGTGCAGAAATGGGATGGAAA	CAACGTCCCTTGGCTTATGCT
ADIPOQ	AAGGAGATCCAGGTCTTATTGGTC	CACACTGAATGCTGAGCGGT
PCK1	ACGGTTATCGTCACCCAAGAG	TACATGGTGCGACCTTTCATGC
SLC2A	CGTCGGGCTTCCAACAGATA	CACCGCAGAGAACACAGCAA
CD36	TGGAGCATTTGATTGAAAAATCCTT	ACAAGCTCTGGTTCTTATTCACA
LPL	TGGAGGTACTTTTCAGCCAGGAT	TCGTGGGAGCACTTCACTAGCT
LIPE	AGCCACGATGGGTGGAATG	CTTGAGGCTGTATCCTGGTAGT
ATGL	GGGCCAGACCCAGCTTC	CCAGGCCTCTGTGAGCC
ABCG1	TGTTCGCGGCCCTCAT	CCTTCAGGCTGTACCAGTAGTTC
